# Scaled Deposition of Ti_3_C_2_*T*_*x*_ MXene on Complex Surfaces: Application Assessment as Rear Electrodes for Silicon Heterojunction Solar Cells

**DOI:** 10.1021/acsnano.1c08871

**Published:** 2022-02-09

**Authors:** Erkan Aydin, Jehad K. El-Demellawi, Emre Yarali, Faisal Aljamaan, Simone Sansoni, Atteq ur Rehman, George Harrison, Jingxuan Kang, Abdulrahman El Labban, Michele De Bastiani, Arsalan Razzaq, Emmanuel Van Kerschaver, Thomas G. Allen, Omar F. Mohammed, Thomas Anthopoulos, Husam N. Alshareef, Stefaan De Wolf

**Affiliations:** † KAUST Solar Center (KSC), Physical Sciences and Engineering (PSE) Division, King Abdullah University of Science and Technology (KAUST), Thuwal 23955-6900, Kingdom of Saudi Arabia; ‡ Physical Sciences and Engineering (PSE) Division, King Abdullah University of Science and Technology (KAUST), Thuwal 23955-6900, Kingdom of Saudi Arabia

**Keywords:** uniform coatings, textured surfaces, cost-effective electrodes, large-area devices, industrial-size MXene

## Abstract

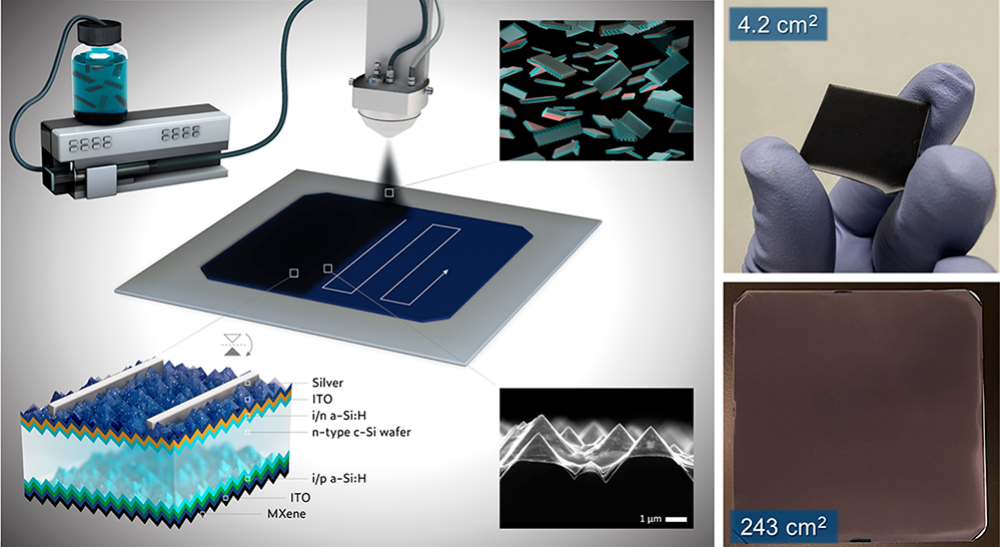

Two-dimensional transition metal carbides (MXenes) are of great interest as electrode materials for a variety of applications, including solar cells, due to their tunable optoelectronic properties, high metallic conductivity, and attractive solution processability. However, thus far, MXene electrodes have only been exploited for lab-scale device applications. Here, to demonstrate the potential of MXene electrodes at an industry-relevant level, we implemented a scalable spray coating technique to deposit highly conductive (*ca*. 8000 S/cm, at a *ca*. 55 nm thickness) Ti_3_C_2_*T*_*x*_ films (*T*_*x*_: surface functional groups, *i*.*e*., −OH, −O, −F) *via* an automated spray system. We employed these Ti_3_C_2_*T*_*x*_ films as rear electrodes for silicon heterojunction solar cells as a proof of concept. The spray-deposited MXene flakes have formed a conformal coating on top of the indium tin oxide (ITO)-coated random pyramidal textured silicon wafers, leading to >20% power conversion efficiency (PCE) over both medium-sized (4.2 cm^2^) and large (243 cm^2^, *i*.*e*., industry-sized 6 in. pseudosquare wafers) cell areas. Notably, the Ti_3_C_2_*T*_*x*_-rear-contacted devices have retained around 99% of their initial PCE for more than 600 days of ambient air storage. Their performance is comparable with state-of-the-art solar cells contacted with sputtered silver electrodes. Our findings demonstrate the high-throughput potential of spray-coated MXene-based electrodes for solar cells in addition to a wider variety of electronic device applications.

## Introduction

The photovoltaic (PV) industry is currently dominated by crystalline silicon (c-Si)-based solar cells, taking a market share of over 95%.^1^ Among these, silicon heterojunctions (SHJs) have demonstrated notable success with power conversion efficiency (PCE) values exceeding 25% in a two-side contacted layout.^2^ SHJ solar cells rely on stacks of intrinsic and doped hydrogenated amorphous silicon (a-Si:H), employed as passivating contacts.^2,3^ Monofacial SHJ solar cells may utilize full-area metallization, typically a 200–300 nm thick sputtered silver (Ag) film, at the rear side of the device, deposited onto transparent conductive oxides (TCOs). With this design, less-conductive TCOs (and hence higher-transparency TCOs) can be employed, which can enhance light trapping of long-wavelength photons in the device while guaranteeing efficient charge collection with a high fill factor (FF).^4,5^ As for the front side, printed Ag fingers and busbars are the prevalent options for the grid metallization for most c-Si technologies, including SHJ devices. Cost-wise, the total Ag consumption is the key driver of the SHJ cell price, which may be affected by the volatility in the Ag market price.^6^ Therefore, considering the increasingly large-scale deployment of PV and scarcity of Ag, relying on Ag contacts may constrain the production capacity of c-Si solar cells in the long term.^7^ To date, the c-Si solar cell industry has exerted several efforts to minimize Ag usage, at either the front or rear sides, by utilizing different printing patterns, stringing schemes, or device shingling or through implementing alternative metallization materials, *e*.*g*., electroplated copper (Cu) and aluminum (Al).^8−10^ However, the large-scale deployment of these alternative electrodes encounters several roadblocks, including low throughput and yield, cost/processing complexity, and potentially large volumes of metal-contaminated waste.^11^ Hence, it is vital to seek highly conductive and less costly nonclassical metallic electrodes with high abundance, easy processability, and low toxicity. In this context, two-dimensional (2D) layered materials, *e*.*g*., graphene,^12^ have recently emerged as an appealing alternative for the prevailing classical metal electrodes (Ag, Cu, Al, *etc.*), thanks to their high conductivity, ease of processing, relatively low cost, and long-term stabilities.

**Figure 1:**
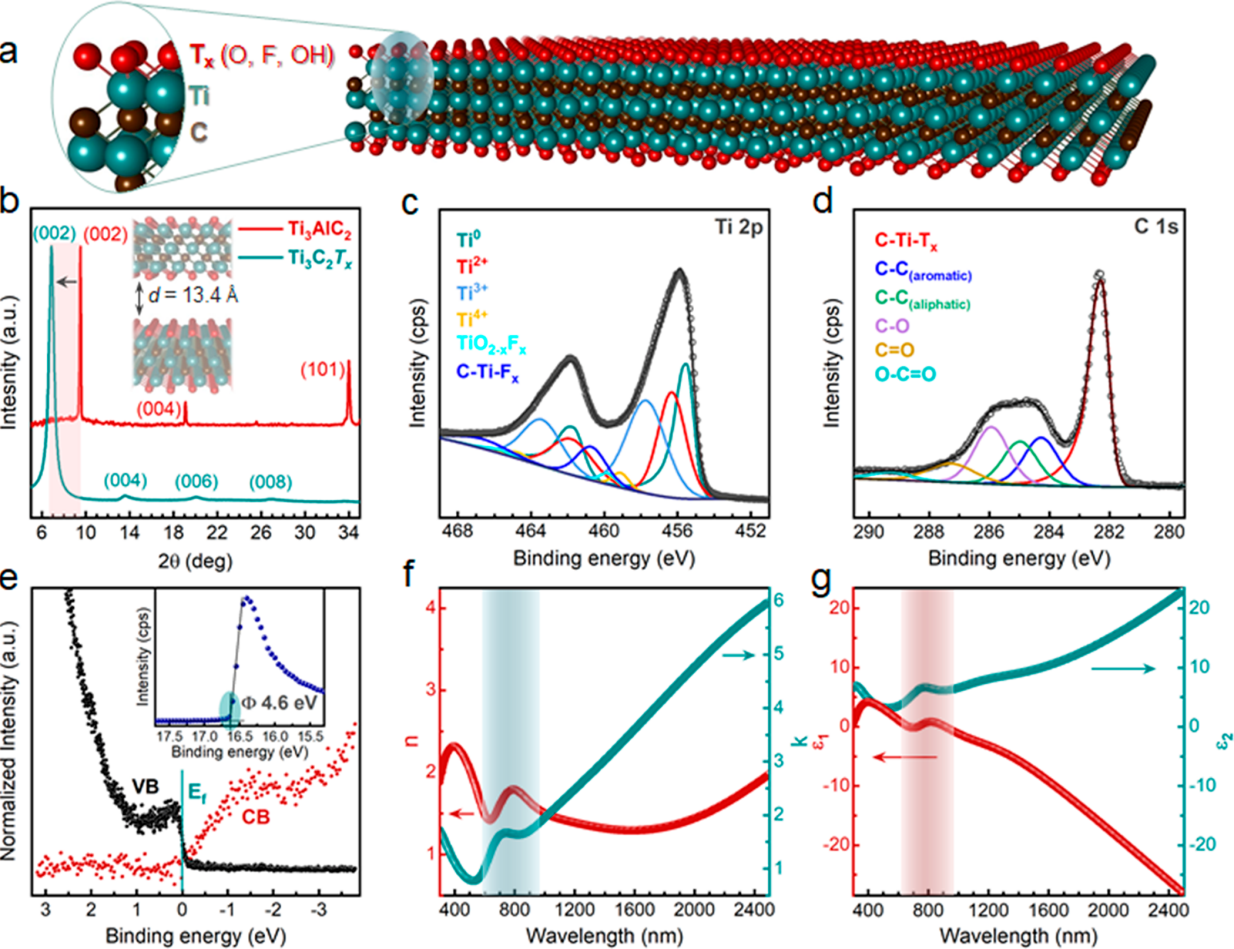
(a) Schematic illustration of a Ti_3_C_2_*T*_*x*_ monolayer, where *T*_*x*_ denotes the surface-terminated functional groups (−O, – OH, −F). (b) XRD spectra (vertically displaced for clarity) of Ti_3_AlC_2_ MAX (top) and Ti_3_C_2_*T*_*x*_ MXene (bottom). (c and d) High-resolution XPS spectra of Ti 2p and C 1s core levels, respectively. (e) Density of states (DOS) at the Fermi level (*E*_f_) in occupied (UPS, black) and unoccupied (IPES, red) spectra of Ti_3_C_2_*T*_*x*_. Inset: UPS secondary electron cutoff (SECO) spectrum along with the extrapolated work function (*i*.*e*., 4.6 eV) of Ti_3_C_2_*T*_*x*_. (f and g) Optical constants (*n*, *k*) and complex permittivity (ε_1_, ε_2_) of a *ca*. 57-nm-thick Ti_3_C_2_*T*_*x*_ spray-coated film, respectively. The shaded regions denote the onset of the characteristic plasmonic absorption of Ti_3_C_2_*T*_*x*_.

Among the existing 2D materials, the fast-growing family of MXenes (*i*.*e*., transition metal carbides, nitrides, or carbonitrides) stands out with their unique set of widely tunable properties.^13,14^ MXenes are typically obtained by removing the “A” layer from their parent ternary MAX phases, where M denotes an early transition metal, A is an element of group 13 or 14, and X is C, N, or both. The general formula defining MXenes is M_*n*+1_X_*n*_*T*_*x*_, where M and X are similar to those of the parent MAX phase, and *T*_*x*_ represents the surface-terminated species (−OH, −O, −F).^15−36^ Since their first report in 2011,^18^ MXenes have been broadly explored for various device applications, including transistors,^17^ photodetectors,^19,20^ sensors,^21^ electromagnetic interference shielding,^22^ solar trackers,^23^ and solar cells.^24^ Most notably for the latter, MXenes were mainly exploited because of their unique optoelectronic properties.^25,26^ Nevertheless, their use as electrodes or charge-selective contacts has outweighed their use as photoactive materials on account of their high electrical conductivity and tailorable work function.^27−29^ More specifically, Ti_3_C_2_*T*_*x*_ (Figure 1a), *i*.*e*., the most mature MXene family member, was deemed eligible to be used as an electrode material for solar cells owing to its superior metal-like conductivity.^15,16,30^ For instance, Agresti *etal*. utilized Ti_3_C_2_*T*_*x*_ in perovskite solar cells (PSCs) to tune the work function at the interfaces for improved charge extraction.^24^ On the other hand, Yang *etal*. managed to enhance electron collection in SnO_2_ electron-selective contacts by mixing them with Ti_3_C_2_*T*_*x*_ MXene.^31^ Again, Yang *etal*. succeeded in improving the performance of PSCs using UV-ozone-treated Ti_3_C_2_*T*_*x*_, where an additional layer of oxide-like Ti–O bonds was formed on the surface of the MXene flakes as a result of the UV-ozone treatment.^32^ Apart from PSCs, Yu *etal*. have efficiently tuned the work function of Ti_3_C_2_*T*_*x*_ and applied it to nonfullerene organic solar cells as electron- and hole-selective contacts.^33^ As for c-Si solar cells, Yi *etal*. have recently shown that Ti_3_C_2_*T*_*x*_ could be used as charge-selective contacts, albeit with only 11% efficiency so far.^34^ Also, Fu *etal*. have demonstrated an 11.5%-efficient c-Si solar cell by utilizing highly conductive Ti_3_C_2_*T*_*x*_ MXene as an electrode.^35^ Although these investigations shed light on the potential of using MXene electrodes in solar cell applications, replacing classical metallic contacts for high-efficiency solar cells with scalable techniques is yet to be demonstrated.

Here, we exploit Ti_3_C_2_*T*_*x*_ MXene films as rear electrodes for monofacial SHJ solar cells, aiming to replace Ag rear contacts, and investigate the applicability of MXenes as electrodes for large-area electronic devices. Taking advantage of their solution processability and hydrophilicity, we opted for spray coating to directly deposit the Ti_3_C_2_*T*_*x*_ flakes on the backside of solar cells. Even though it is considered the most advantageous method for MXene-based device fabrication,^36^ spray coating of MXenes has hitherto been applied only on a small scale and primarily by hand-spraying techniques. Here, utilizing an automatized ultrasonic spray coater enabled us to spread the Ti_3_C_2_*T*_*x*_ flakes over the typical micron-scale pyramidal textured backside of SHJ solar cells with highly uniform coverage. Eventually, the fabricated MXene rear contacts have yielded high-efficiency SHJ solar cells with >20% PCE values, at MXene thicknesses of only *ca*. 200 nm. We also demonstrate how the automated spray-coating system allows for fine thickness control and homogeneity over large areas, bringing industrial relevance to the process. As a result, our large-area MXene-based devices maintain their PCE with a scaling factor of ∼58 (from 4.2 to 243 cm^2^). Overall, we demonstrate the potential use of Ti_3_C_2_*T*_*x*_ MXene as an electrode for large-area, high-throughput industrial electronic applications.

## Results and Discussion

In this work, Ti_3_C_2_*T*_*x*_ MXene was exfoliated from its parent MAX phase (*i*.*e*., Ti_3_AlC_2_) by selectively removing the Al layer in a fluoride- and chloride-containing etching bath. As described in the Methods, few- and multilayered Ti_3_C_2_*T*_*x*_ flakes were obtained through a well-controlled Li^+^-based delamination process, with a selectively tuned average flake size of approximately 2 μm, knowing that smaller flakes are more prone to oxidation.^36^Figure S1 schematically illustrates the synthesis pathway of the Ti_3_C_2_*T*_*x*_ flakes dispersed in deionized (DI) water. The crystal and stoichiometric quality of our Ti_3_C_2_*T*_*x*_ flakes were respectively studied using X-ray diffraction (XRD) and X-ray photoelectron spectroscopy (XPS). The crystallinity of the Ti_3_C_2_*T*_*x*_ films spray-coated on glass substrates is demonstrated in Figure 1b, showing all the diffraction peaks of a typical Ti_3_C_2_*T*_*x*_ pattern in the 5–35° range. Following the etching process, the characteristic (002) peak of Ti_3_AlC_2_ was shifted from 2θ = 9.5° down to 6.86° for Ti_3_C_2_*T*_*x*_, indicating an increase in the interlayer spacing (*d*) (inset of Figure 1b). The broadening and shift in the characteristic (002) peak are attributed to the substitution of the Al layers with the surface-terminating groups (*T*_*x*_), resulting from the exfoliation followed by the delamination. Meanwhile, the oxidation stability of the synthesized MXene is manifested by the high-resolution XPS spectra of the Ti 2p and C 1s core levels, as demonstrated in Figure 1c,d, respectively. The corresponding XPS survey scan of the studied Ti_3_C_2_*T*_*x*_ sample is depicted in Figure S2. In principle, maintaining a low content of oxidized species is crucial to preserve the intrinsic characteristics of our Ti_3_C_2_*T*_*x*_ MXene. The Ti 2p region of the Ti_3_C_2_*T*_*x*_ spray-coated film, presented in Figure 1c, was fitted utilizing mixed Gaussian–Lorentzian GL(30) products, after background subtraction, with a metallic asymmetry arising from the C-Ti-*T*_*x*_ component. The corresponding fitting parameters are discussed in the SI and summarized in Table S1. The Ti 2p_3/2_ components were located at 455.5, 456.3, 457.7, 459.1, 459.7, and 460.6 eV, respectively, assigned to the Ti oxidation states, *i*.*e*., Ti^0^ (Ti-C),^37^ Ti^2+^, Ti^3+^,^37,38^ Ti^4+^ (titania, TiO_2_),^39^ fluorinated titania (TiO_2–*x*_F_*x*_), and C-Ti-F_*x*_.^38^ The Ti^2+^ and Ti^3+^ oxidation states are related to Ti bonded to C and O, *i*.*e*., Ti_3_C_2_O_*x*_ and Ti_3_C_2_(OH)_*x*_ species.^38^ They could also be associated with Ti bonded to OH with an overlayer of adsorbed H_2_O_(ad)_. Although the oxidation of MXene is inevitable, especially in air, the amount of semiconducting TiO_2_ species (the yellow spectrum in Figure 1C) is minimal (relatively 3% less than the Ti-C component), rendering our MXene flakes highly conductive. Figure 1d displays the C 1s core level with its asymmetric peak shape, which is deconvoluted into six main components, *i*.*e*., C-Ti-T_*x*_, C–C_(aromatic)_, C–C_(aliphatic)_, C–O, C=O, and O–C=O^38,40^ located at 282.3, 284.3, 284.9, 285.9, 287.3, and 289.3 eV, respectively.^41,42^ A summary of the corresponding fitting parameters of the C 1s region is provided in Table S2.

The metal-like behavior of our synthesized Ti_3_C_2_*T*_*x*_ is demonstrated by the low sheet resistivity (about 30 Ω/sq) exhibited by a 57-nm-thick spray-coated MXene film. Furthermore, the corresponding Hall-effect measurements, conducted on the same films, have revealed a carrier concentration of *ca*. 7 × 10^22^ cm^–3^ and a bulk resistivity of ca. 1.36 × 10^–4^ Ω·cm (average of three films coated on glass), only 1 order of magnitude higher than that of sputtered Ag (*i*.*e*., 1 × 10^–5^ Ω·cm), confirming the metallic behavior of our Ti_3_C_2_*T*_*x*_ MXene films. The fingerprint of this metal-like conductivity is also demonstrated by the XPS valence band (VB) edge of spray-coated MXene films, as shown in Figure S3. The distinctive step function at the Fermi level (*E*_f_) is indicative of a large density of occupied electronic states at the Fermi edge, which is a typical metallic (Fermi–Dirac) step feature and is qualitatively comparable with that of Ag foil (Figure S3).

**Figure 2:**
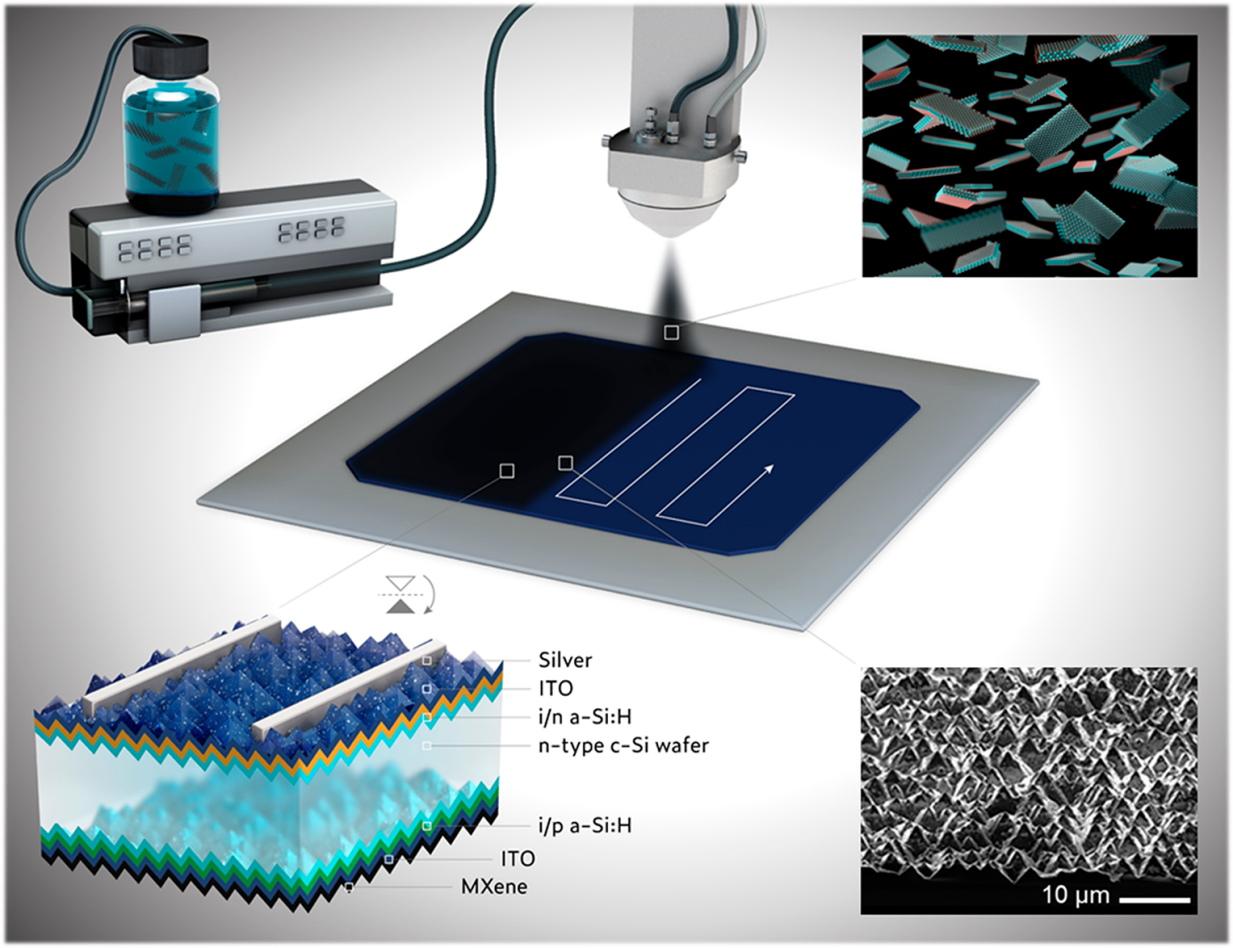
Schematic representation of the automated spraying apparatus of large-scale deposition of Ti_3_C_2_*T*_*x*_ flakes as the back electrode for SHJ solar cells. Insets: (Bottom-left) Corresponding layer-by-layer structure of an SHJ solar cell. (Bottom-right) Tilted top-view SEM micrograph of the Ti_3_C_2_*T*_*x*_ flakes covering the ITO-coated pyramidal textured surface of SHJ solar cells.

The occupied and unoccupied states of the Ti_3_C_2_*T*_*x*_ surface were respectively probed using ultraviolet photoelectron spectroscopy (UPS) and inverse photoemission spectroscopy (IPES). Both UPS and IPES (Figure 2e) evidence the presence of states at the *E*_f_, in line with the exhibited metallic character of our MXenes. Such a metallic behavior is the origin of the asymmetric line shape of the Ti-C component, as shown in the high-resolution XPS spectrum of the Ti 2p region, which is related to the photoelectron energy loss mechanism coupled to the density of states (DOS) at *E*_f_. The inset of Figure 2e displays an extrapolated work function (Φ) of 4.6 eV for our Ti_3_C_2_*T*_*x*_, which accords well with previous reports.^43^ In principle, the work function of MXenes is strongly dictated by the dipole moments between the transition metal (*e*.*g*., Ti) and the surface-terminated species (*T*_*x*_).^22,27,44^ Hence, precisely controlling the synthesis conditions is imperative for maintaining reproducible batch-to-batch work function values.

Next, we investigated the optoelectronic properties of the MXene flakes using spectroscopic ellipsometry. Figure 1f shows the optical constants *n* (refractive index) and *k* (extinction coefficient) of a *ca*. 57-nm-thick Ti_3_C_2_*T*_*x*_ film (spray-coated on an ITO/c-Si stack) in the spectral range of 300–2500 nm. The corresponding fitting parameters are discussed in the SI. The dispersion of the dielectric permittivity of the Ti_3_C_2_*T*_*x*_ film is displayed in Figure 1g, where ε_1_ and ε_2_ represent the real and the imaginary parts of the dielectric constant. The shaded regions mark the characteristic out-of-plane surface plasmons (SPs) localized at the surface of the MXene flakes with a resonant frequency at 760 nm.^30^ The real part of the permittivity (ε_1_) stays negative at longer wavelengths, indicating a pronounced plasmonic absorption, which is in line with the increase in the *k* values at longer wavelengths. For less metallic materials, the onset of such plasmonic absorption is typically featured at lower frequencies in the infrared and is highly dependent on the free carrier concentration.^45,46^ However, given the metal-like high free carrier concentration in Ti_3_C_2_*T*_*x*_, its plasmonic band emerges within the visible spectral regime.

To examine the above-mentioned metal-like behavior of Ti_3_C_2_*T*_*x*_ films and demonstrate the application of MXenes as electrodes on large-area electronic devices, we opted to use them as the rear electrodes for large-area SHJ solar cells. Refer to the Methods section for the fabrication details of the SHJ solar cells. For comparison, we contacted the rear side of the studied SHJs with three different rear electrode stacks, respectively consisting of indium tin oxide (ITO) (control sample), ITO/Ag, and ITO/Ti_3_C_2_*T*_*x*_. To spray-coat the MXene rear electrodes, we utilized an ultrasonic automated spraying system at moderate substrate temperatures (150–180 °C), which is critical to avoid the degradation of the SHJ cells. Generally, spraying at higher temperatures may promote the effusion of H from the a-Si:H passivation stacks, resulting in low open-circuit voltage (*V*_oc_) values.^47^Figure 2 provides a schematic illustration of the automated spraying apparatus we used to spray-coat large-area MXene contacts (4.2 and 243 cm^2^) in several minutes at a 20 nm/min deposition rate. More details about the spraying protocol are provided in the Methods section. The concentration of all the used Ti_3_C_2_*T*_*x*_ suspensions (top-left inset in Figure 2) was fixed at 1.35 mg/mL. In this study, we used double-side random pyramidal textured SHJ cells, typical for commercial solar cells, as illustrated in the sketched layer-by-layer structure (bottom-left inset of Figure 2).

**Figure 3:**
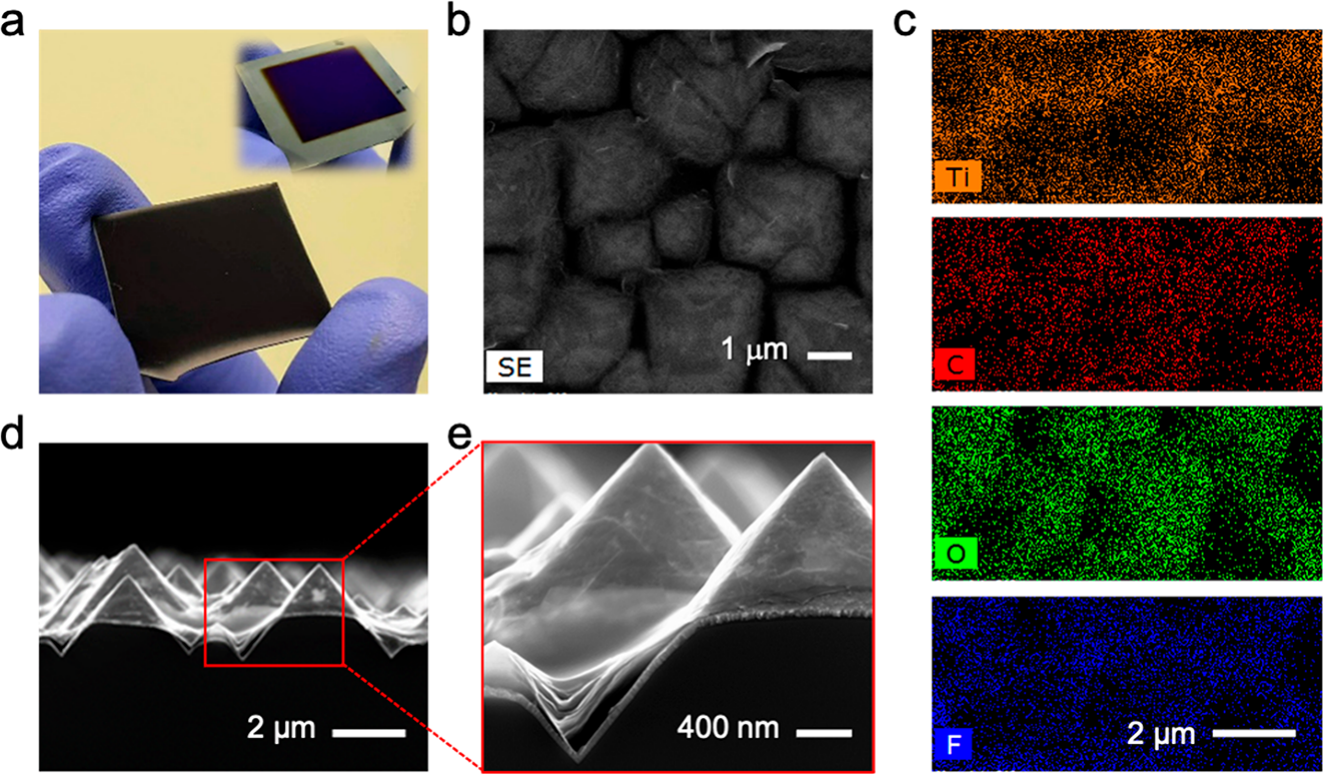
(a) Rear side of a Ti_3_C_2_*T*_*x*_-contacted SHJ solar cell (4.2 cm^2^ in area). Inset: Rear side of the SHJ device before spraying Ti_3_C_2_*T*_*x*_. (b) Top-view secondary electron (SE) SEM micrograph of a Ti_3_C_2_*T*_*x*_ film sprayed on the textured interface of an SHJ cell and (c) corresponding EDS elemental maps showing the homogeneity of Ti (orange), C (red), O (green), and F (blue) across the textured surface. (d and e) Cross-section SEM micrographs of a 57-nm-thick spray-coated Ti_3_C_2_*T*_*x*_ rear contact, showing the uniform surface coverage of the MXene flakes on the textured surface of the SHJ cell.

The Ti_3_C_2_*T*_*x*_ rear contacts were spray-coated for thicknesses between 50 and 400 nm (sputtered Ag was around 250 nm) by controlling the number of spray-coating cycles from 40 to 260. Although spray coating allows for fast and uniform coating on large-area planar surfaces, this could be challenging on more complex geometries and textured surfaces, like in the case of random-pyramidal textured SHJ cells. Hence, optimizing the processing parameters is essential, including dispersion concentration, spraying flow rate, substrate temperature, and the distance between the nozzle and the substrate. Following the spray coating, we verified the uniformity of the surface coverage on the backside of SHJ devices using scanning electron microscopy (SEM). The bottom-right inset in Figure 2 displays a tilted top-view SEM micrograph of an MXene-coated pyramidal textured SHJ device, showing uniform surface coverage across the backside. Figure 3a shows the backside of an actual 4.2 cm^2^ solar cell before and after spray coating the MXene onto the rear ITO. The surface uniformity was further confirmed using energy-dispersive X-ray spectroscopy (EDS) in conjunction with SEM to scan the elemental mapping over the spray-coated surface. Figures 3b,c and S4 demonstrate the homogeneous distribution of all characteristic elements comprising the MXene flakes, *i*.*e*., Ti, C, O, and F, along with the elemental distribution of the underneath ITO-coated silicon. The cross-section SEM micrographs demonstrate the uniform coverage of our Ti_3_C_2_*T*_*x*_ flakes over the facets of backside pyramids, as shown in Figure 3d,e. The uniform coverage over the pyramids, either on the facets or in the valleys, is notable for solution-processable contacts made of 2D flakes, which is rarely achieved with solution-based techniques.^48^

**Figure 4:**
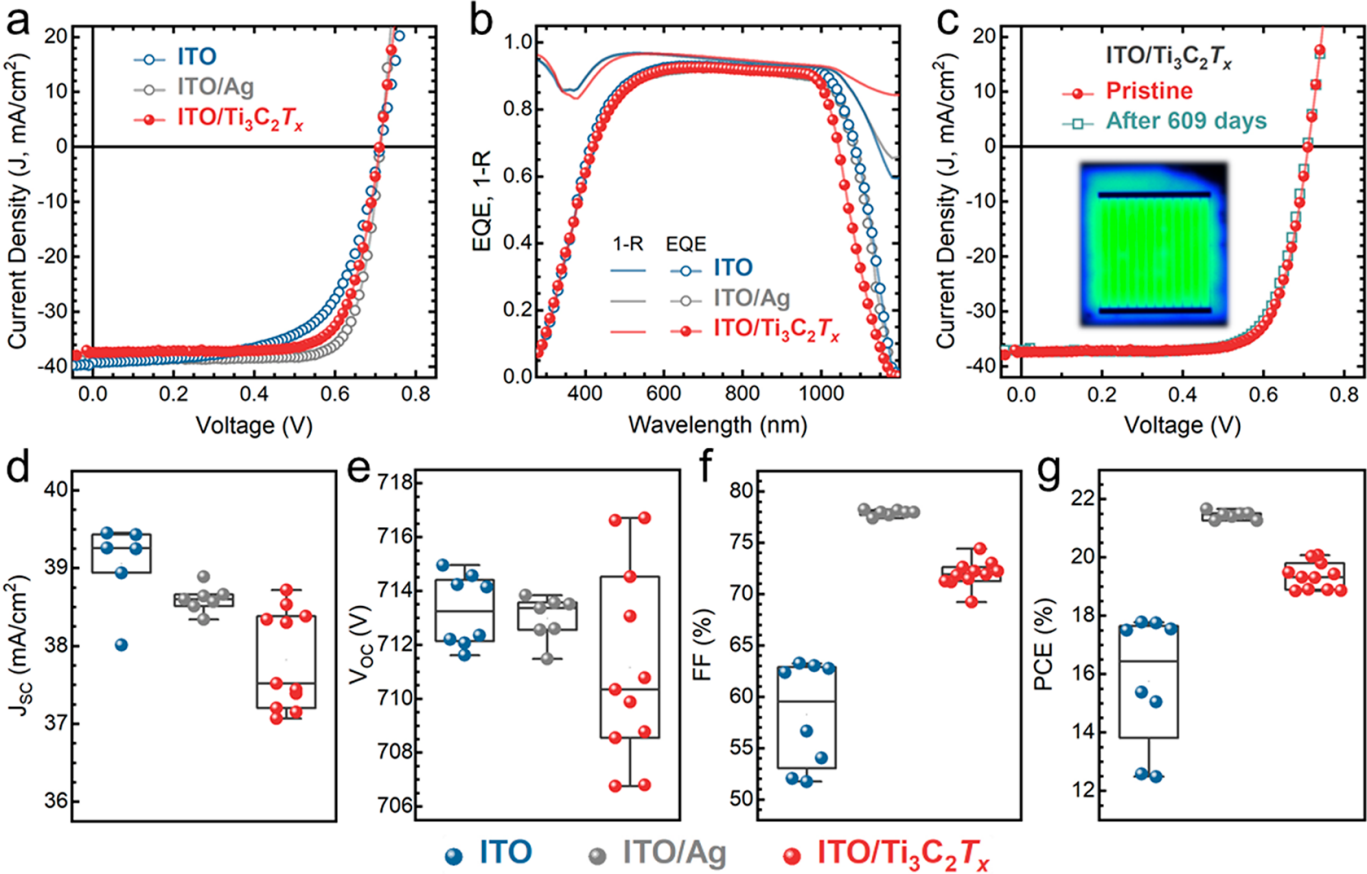
(a) *J–V* characteristics of SHJ solar cells (4.2 cm^2^ in area) with different rear electrode stacks: ITO only (blue, 100 nm), ITO/Ag (gray, 100/250 nm), and ITO/Ti_3_C_2_*T*_*x*_ (red, 100/200 nm). (b) Corresponding EQE and 1-R spectra. (c) *J–V* characteristics of a 19.8%-efficient Ti_3_C_2_*T*_*x*_-contacted SHJ cell measured at fabrication time (red) and after 609 days (green) with almost no degradation. Inset: PL image indicating no degradation after tapping the front side of the cell while spraying the MXene. (d–g) Statistical summary of device characteristics (*J*_SC_, *V*_OC_, FF, and PCE) of all the tested SHJ solar cells.

To realize the optimum thickness of our MXene rear contacts on SHJ cells, we fabricated an array of 4.2 cm^2^ devices by spraying the MXene-containing DI dispersion, as shown in Figure 2, using different numbers of cycles (from 40 to 260) for adjusting the thicknesses of the MXene layers. We then tested the device performance of all the spray-coated 4.2 cm^2^ solar cells and found that the PCE plateaued at 19.3% (±0.5) after 60 spraying cycles (*i*.*e*., about 85 nm). Table S4 summarizes the overall performance of all the 4.2 cm^2^ MXene-contacted SHJ solar cells for different spraying cycles. It is important to note here that the work function of our Ti_3_C_2_*T*_*x*_ (*i*.*e*., 4.6 eV) was sufficient to create an ohmic contact at the MXene/ITO interface and is on par with the work function of Ag (4.2 to 4.6 eV, based on the amount of surface oxide). Looking at Table S4, spraying Ti_3_C_2_*T*_*x*_ for 140 cycles (200 nm, *i*.*e*., 50 nm less than sputtered Ag) has led to MXene-contacted SHJ solar cells with a PCE of 19.8% along with an FF of 74.4% (the highest attained FF among the other utilized spraying cycles in our work). As a result, we further optimized the other spraying conditions, particularly the flow rate, while fixing the number of spraying cycles at 140. We obtained a PCE of 20.1% for Ti_3_C_2_*T*_*x*_-contacted SHJ solar cells, compared to 21.6% for Ag-contacted control devices. Figure 4a depicts the *J–V* characteristics of three devices, each contacted with a different rear electrode stack. Without any metallic contact to the rear ITO, the FF and PCE of the devices are much lower than the contacted cells. Not surprisingly, Ag-contacted cells provide better back reflection than MXene-contacted devices, mainly due to the high absorption cross-section of Ti_3_C_2_*T*_*x*_ films (Figure 4b). Therefore, the near-infrared (NIR) response of the Ag-contacted devices is relatively higher than that of MXene-based devices, which is also verified by the external quantum efficiency (EQE) measurements (Figure 4b). This reduction in the back reflection originates from the localized NIR plasmonic absorption losses at the rear MXene contacts, as proven by the negative dispersion of the real part of the dielectric permittivity provided in Figure 1f,g. This loss can be minimized by employing thicker TCOs (∼100–200 nm) at the rear with sufficiently low carrier densities to avoid free carrier absorption, optically displacing the MXene from the c-Si surface.^5,49^ In this case, rear side TCO serves as an optical spacer by shifting the metal surface plasmon to shorter wavelengths that are completely absorbed within the first few microns of the c-Si substrate.^47^ Additionally, rear side TCO increases the refractive index mismatch between c-Si and the rear electrode for improving the internal rear reflectance. Other possibilities include using distributed Bragg reflectors,^50^*e*.*g*., using stacks of TCOs with alternating refractive indices, or integrating the rear side diffraction gratings^51^ in place of the usual random pyramidal texture.

We also monitored the stability of our Ti_3_C_2_*T*_*x*_-contacted device performance after 20+ months of ambient air storage. Figure 4c demonstrates the corresponding *J–V* characteristics of one of our MXene-based devices (19.8% efficient) measured at fabrication time and after 609 days, with a slight degradation given that the PCE only dropped to 19.5%. The corresponding SEM micrographs (Figure S5) show no morphological deterioration in the MXene films after this prolonged ambient storage. The 609-day-old performance of other MXene-contacted devices was also recorded and statistically compared with their initial performance (Figure S6). Interestingly, the measured devices have retained between 98.5% and 99.7% of their initial PCE values. Although Ag-contacted SHJ solar cells can often remain unchanged for a long time, such long-lasting ambient stability for SHJs with nonclassical metal contacts is notable considering the well-known oxidation sensitivity of untreated MXene films.^52,53^ Also, the shelf life of these devices can be prolonged if they are encapsulated, as is usually done with standard c-Si modules. To observe the reproducibility of the synthesis and spraying quality of Ti_3_C_2_*T*_*x*_, we fabricated three subsequent batches of MXene-contacted devices. Figure 4d–g display a statistical summary of the overall performance of all the fabricated SHJ solar cells. The statistically broader variation in the performance of the Ti_3_C_2_*T*_*x*_-contacted cells compared to the Ag-contacted ones is solely related to the induced damage during sample handling and processing, where the cells had to be fixed on the holder using tapes, causing partial damage to the devices. In the future, vacuum chucks and physical fixtures will be used to minimize such surface deteriorations.

**Figure 5:**
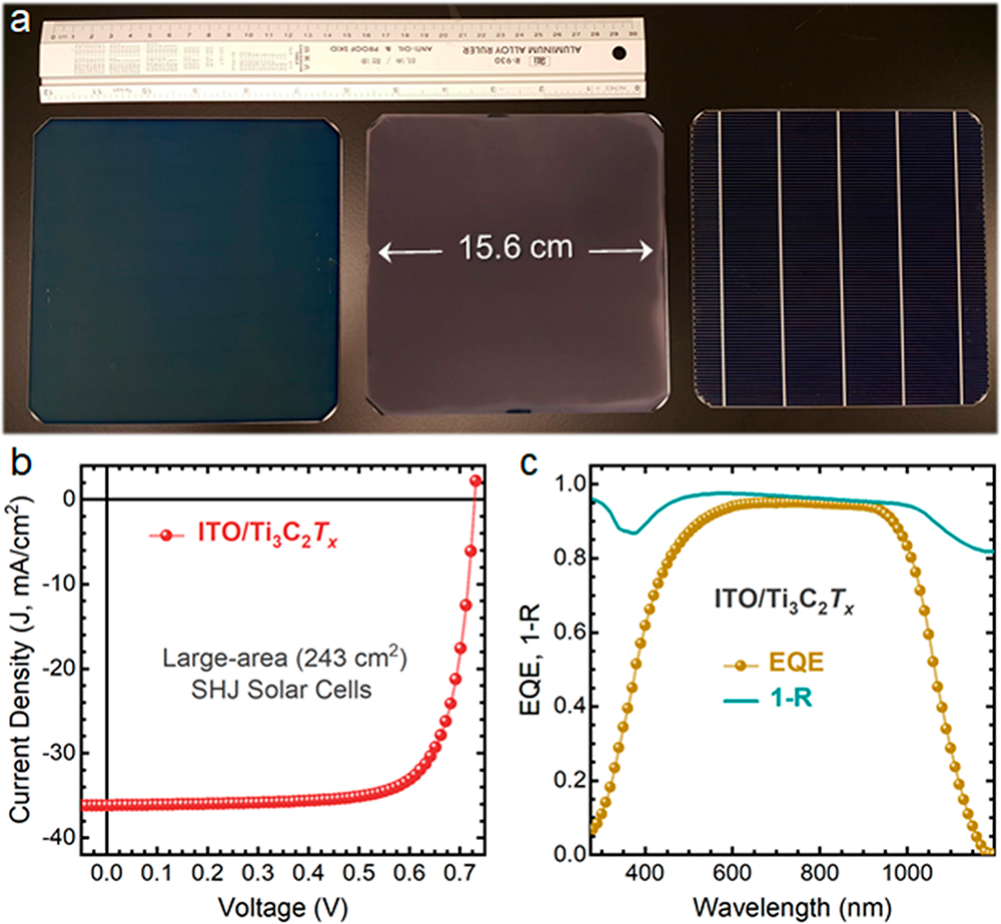
(a) Photograph of the two sides of a large-area (6-in.) SHJ solar cell before and after spraying (140 cycles) the Ti_3_C_2_*T*_*x*_ MXene (200 nm thick) on the rear side (left and middle, respectively). The SHJ cells are juxtaposed with a 30-cm-long ruler as a reference scale. (b) *J–V* characteristics of the Ti_3_C_2_*T*_*x*_-contacted SHJ solar cell (243 cm^2^ in area). (c) Corresponding EQE and 1-R spectra of the same solar cell in (b).

As a proof of concept for their scalability, we spray-coated Ti_3_C_2_*T*_*x*_ contacts over industrial-grade 6 in. (243 cm^2^ in area) SHJ cells instead of Ag contacts (Figure 5a). Markedly, *J–V* measurements performed on a copper back contacted stage under standard testing conditions (AM1.5G spectrum, 1000 W m^–2^, 25 °C) have revealed that the performance of the MXene contacts retains their PCE with a scaling factor of 58 (from 4.2 to 243 cm^2^) (Figure 5b,c). We note that the utilization of thinner silicon wafers (around 130 μm) has yielded higher *V*_OC_ values for the 6 in. devices than those obtained for the 4.2 cm^2^ devices (fabricated with 250-μm-thick Si wafers). Interestingly, our large-area MXene-contacted SHJ solar cells have a series resistance of 2.0 Ω cm^2^, on par with Ag-contacted devices (1.9 Ω cm^2^).

An overview of the device performance of a selection of 4.2 and 243 cm^2^ solar cells contacted with the three tested rear electrode stacks (*i*.*e*., ITO-only, ITO/Ag, and ITO/Ti_3_C_2_*T*_*x*_) is summarized in Table 1. The comparable PCE values of the Ag- and MXene-contacted SHJ devices highlight the applicability of MXene-based electrodes on large-area electronic devices. Given the nascent research stage on the ever-expanding family of MXenes, its vast stoichiometric flexibility could lead to subsequent development in terms of device performance or stability. For instance, the work function of MXenes is known to be sensitive to any change in the surface dipole moments arising between the transition metal (*e*.*g*., Ti, V, Nb, Mo) and the surface-terminated groups. Thus, any modification to the rich surface chemistry of any MXene (*e*.*g*., Ti_3_C_2_*T*_*x*_, V_2_C*T*_*x*_, Nb_2_C*T*_*x*_) would alter its work function, resulting in adjustable barrier heights and saturation current densities at the interface between that of MXene and the underlying layers, leading to tunable *V*_OC_ and better control over device performance. Likewise, the onset of the parasitic plasmonic absorption losses at the Ti_3_C_2_*T*_*x*_ electrodes depends on the free carrier concentration, which is highly affected by the surface-terminated functional groups (*T*_*x*_). Thus, by controlling the relative amounts of these surface species, we can mitigate the above-mentioned reduction in the back reflection at the MXene electrodes, leading to an enhanced EQE. Furthermore, given their superior solution processability, there is still ample room to improve the conditions of spraying MXene dispersions (*e*.*g*., flow rate, concentration, working distance, temperature), leveraging its contact resistivity and resulting in higher FFs.

**Table 1:** Performance Overview for a Selection of Our Best Performing Fabricated SHJ Solar Cells with Different Electrodes

electrode stack	area (cm^2^)	wafer thickness (μm)	*V*_OC_(mV)	*J*_SC_(mA/cm^2^)	FF (%)	PCE (%)
ITO	4.2	∼250	712.1	33.9	51.7	12.5
ITO/Ag			712.5	38.9	78.3	21.7
ITO/Ti_**3**_C_2_*T*_*x*_			716.7	38.3	73.1	20.0
ITO/Ag	243	∼180	731.8	37.5	75.6	20.7
ITO/Ti_3_C_2_*T*_*x*_			726.3	36.0	73.3	19.3

## Conclusion and Outlook

In this study, Ti_3_C_2_*T*_*x*_ MXene was exploited as a rear full-area electrode for SHJ solar cells to demonstrate the application of MXenes as a scalable electrode material for large-area electronic devices. For this, we first synthesized dispersions of relatively large freestanding Ti_3_C_2_*T*_*x*_ flakes (≥2 μm in size), knowing that smaller ones are more susceptible to faster oxidation rates, which may limit their conductivity. MXene contacts were applied on the backside of SHJ devices utilizing an automated ultrasonic spraying technique, providing a layer-by-layer deposition of the flakes on the random pyramidal textured interfaces. Eventually, we obtained high-quality MXene rear contacts for SHJ solar cells with superior uniformity along the pyramidal textured surface. The PCE attained by Ti_3_C_2_*T*_*x*_-contacted devices (4.2 cm^2^ in area) has exceeded 20% with good reproducibility and prolonged stability for more than 20 months (unencapsulated cells). The PCE of the MXene-contacted devices is only about 1% absolute less than that of devices contacted with Ag rear electrodes. After upscaling, we achieved a PCE of 20% for Ti_3_C_2_*T*_*x*_-based SHJ cells over 6-in. industry-size wafers, commensurate with the Ag-contacted counterparts. These findings mark the potential of the MXenes as scalable electrode materials for several electronic device applications. Besides, the low series resistance attained by the 243 cm^2^ MXene-based SHJs is auspicious for large-area devices contacted with nonclassical metallic contacts. Additionally, employing automated spray coating has allowed us to fabricate MXene electrodes at a fast deposition rate, which can be used for industrial-scale electronic applications.

Apart from SHJ devices, and solar cells in general, the ability to acquire upscaled well-performing MXene contacts on rough surfaces with complex geometries is appealing for various applications, especially if the MXene suspensions can be manufactured at a large scale and low cost. The herein reported processing speed can be increased further by spraying the MXene ink *via* multiple heads to minimize the number of cycles and reach a throughput of thousands of wafers per hour. The feasibility of this process can be the focus of more research on this topic. Ultimately, our study renders MXenes a potential replacement for costly metals, extending their use to other industrially applicable devices. Nonetheless, we note that the stability of these contacts must be carefully examined, for future studies, using standard accelerated testing methods, such as International Electrotechnical Commission (IEC) test protocols. We also note that the solderability of external contacts to the MXene electrodes must be verified for further electronic device integration.

## Methods

### Synthesis of MXene Flakes

Suspensions of Ti_3_C_2_*T*_*x*_ MXene flakes were prepared by selectively etching the Al layer from the parent MAX phase, *i*.*e*., Ti_3_AlC_2_. In a typical experiment, we added 1 g of Ti_3_AlC_2_ powder (<40 μm particle size, Carbon-Ukraine ltd.) to a 10 mL etching bath composed of hydrofluoric acid (HF, VWR Chemicals), hydrochloric acid (HCl, Sigma-Aldrich), and DI water (Milli-Q) at a volume ratio of 3:6:3. The mixture was then stirred at 40 °C for *ca*. 15 h. Afterward, the resultant suspension of exfoliated MXene flakes went through several centrifugation/decantation rounds to attain a pH value of 6–7. For delamination, the exfoliated MXene flakes were intercalated using lithium chloride (LiCl, Sigma-Aldrich). Subsequently, the LiCl-intercalated MXene suspensions were subjected to another round of centrifugation/decantation until a pH value of 6–7 was reached. The size (lateral dimension) of the MXene flakes was carefully controlled by adjusting the centrifugation time and speed. Supernatant solutions of delaminated Ti_3_C_2_*T*_*x*_ flakes, with an averagely larger size (∼2 μm), were collected after longer centrifugation rounds (20+ minutes), yet slower (∼1000 rpm). The final MXene suspensions were stored at *ca*. 3 °C to slow down their inevitable oxidation in water. The synthesis pathway is summarized in Figure S1 in the SI.

### Spray Coating of MXene Films

Ultrasonic spray pyrolysis was implemented using a fully automated Sono-Tek Corporation coating system to spray-coat the Ti_3_C_2_*T*_*x*_ flakes (suspended in DI water at >20 °C) onto different surfaces (*i*.*e*., glass substrates and the ITO-coated rear side of the SHJ cells). Before the deposition of the MXene rear contacts, the front sides of the solar cells (facing down) were covered with Kapton tape. The ITO-coated backsides (facing up) were then exposed to 10 min of UV/ozone treatment to enhance their wettability. During the spraying of the Ti_3_C_2_*T*_*x*_ flakes, the substrate temperature was set at 180 °C. The thicknesses of the films were controlled by the number of passes of the nozzle rastering across the samples while spraying the fine mist of the MXene dispersion. The solution feed rate was kept at 2 mL/min, at a working distance of 13 cm between the substrate and the nozzle.

### Structural Characterization

XRD analysis was conducted using a powder X-ray diffractometer (Bruker D8 Advance) with Cu Kα radiation at a wavelength of 1.5418 Å. The scanning rate was 0.02°/step at 0.5 s/step in the 2θ range of 5–65°. SEM and EDS analyses were performed using a Carl Zeiss AURIGA CrossBeam workstation equipped with a focused ion beam (FIB) column and a Quantax EDS spectrometer from Bruker. In-lens SEM images were taken at an electron high tension (EHT) of 5 kV and a working distance (WD) of 6 and 3 mm for top-view and cross-sectional images. EDS measurements were performed at 10 keV by analyzing secondary-electrons top-view images acquired at 15 kV EHT, 6 mm WD, and 10 000× magnification.

XPS, UPS, and IPES (low-energy) measurements were performed in an interconnected ScientaOmicron UHV system. The system was equipped with a monochromated XM1000 Al Kα X-ray source (λν = 1486.6 eV) and a VUV He lamp (Focus). The photoelectrons were measured with a hemispherical Sphera II EAC125 analyzer and a multi-channeltron detector. Samples were fixed to the Omicron style plates with Ta clips in electrical contact to the analyzer, recorded without charge neutralization. UPS was performed between 10 and 5 eV constant pass energy and XPS at 50 eV for the survey and 15 eV for the high-resolution scans, with an applied −10 V bias. IPES was conducted in isochromatic bremsstrahlung mode in a separate dedicated chamber. A Staib electron source was used between 20 and 28 eV beam energy with 0.25 eV energy spread directed perpendicular to the surface. The sample was biased at +20 V. The emission was detected with a lens assembly and a 254 nm bandpass filter (Semrock) and collected with a Hamamatsu R985 PMT.

### Optoelectronic and Electrical Characterizations

Spectroscopic ellipsometry measurements were performed on MXene films using a VUV-VASE variable-angle spectroscopic ellipsometer (J.A. Woollam M-2000 DI) in the range of 193 to 1690 nm. The ellipsometric data were collected over Ti_3_C_2_*T*_*x*_ MXene films spray-coated onto ITO/c-Si substrates in the spectral region of 300 to 2500 nm, at multiple incident angles from 60° to 75° with a step size of 5°. Transmittance/reflectance measurements were performed using a PerkinElmer Lambda 950 UV/vis–NIR spectrophotometer. Hall-effect and conductivity measurements were conducted at room temperature using a Lake Shore 7700 Hall system in Van der Pauw geometry. Series resistance values are extracted from the *J*–*V* curves.

### Fabrication of Silicon Heterojunction Solar Cells

Small-area (4.2 cm^2^) SHJ solar cells were fabricated on float-zone double-side textured four-inch wafers (TOPSIL, n-doped, resistivity 1–5 Ω·cm, thickness 250–280 μm). The texturing process was performed in an alkaline solution to achieve randomly distributed pyramids. The wafers were subsequently cleaned in RCA-1 and RCA-2 solutions. Large-area (243 cm^2^) SHJ solar cells were fabricated on a pseudosquare CZ double-side textured wafer (Longi, n-doped, resistivity 5–10 Ω·cm, thickness 120–130 μm). A saw-damage-removal process in an alkaline solution was performed before the texturing process. Prior to the PECVD depositions, the wafers were dipped in a 5% HF solution to remove the RCA-2 oxide layer. The intrinsic, n- and p-type amorphous silicon layers were deposited in a PECVD cluster (Indeotec Octopus II) with silane, hydrogen, phosphine, and trimethylboron as input gases. The front and back contacts were formed in the PVD part of the Octopus cluster by sequentially sputtering ITO (front and back 75 and 100 nm, respectively) and Ag (250 nm), where required. All depositions were performed at temperatures of ≤200 °C. For both small-area and large-area SHJ, the top contact is concluded with a silver grid deposited by screen-printing and annealed at 200 °C.

### Characterization of SHJ Solar Cells

*J–V* measurements were performed at 25 °C using a Wavelabs Sinus 220 LED-based solar simulator with an AM 1.5G irradiance spectrum. The *J–V* measurements were performed on the vacuumed copper plate with a voltage sensing pin in the middle and spring-loaded probes on the front. The light intensity was calibrated using Fraunhofer ISE CalLab-certified c-Si solar cells. For small-area cells, the illumination area of the devices was 4.2 cm^2^, which is determined by the laser cut shadow mask coated with matte black paint. The large-area devices were measured without the shadow mask, considering the active area as the wafer area. EQE measurements were performed using PV-Tools LOANA equipment by measuring the temperature from the backside of the wafers, similar to *J*–*V* measurements. All device characterizations were performed in ambient air with an average RH ≈ 50% and without encapsulation.

## Supporting Information Available

Synthesis pathway of Ti_3_C_2_*T*_*x*_, XPS survey scan of Ti_3_C_2_*T*_*x*_ film, valence band XPS spectra of Ti_3_C_2_*T*_*x*_, ellipsometry data analysis, EDS mapping of Ti_3_C_2_*T*_*x*_ film on silicon bottom cell, summary of the device characteristics, SEM, and statistical representation of performance stability (PDF)
