# Supplementary material for: Scaled Deposition of Ti3C2Tx MXene on Complex Surfaces: Application Assessment as Rear Electrodes for Silicon Heterojunction Solar Cells
Source: ACS Nano. 2022 Feb 9;16(2):2419–28. doi: 10.1021/acsnano.1c08871 (PMC8867910; doi:10.1021/acsnano.1c08871)
Supplement: Supplementary file 1 — nn1c08871_si_001.pdf [file nn1c08871_si_001.pdf]

Supporting Information

Scaled Deposition of $Ti_3C_2T_x$ MXene on Complex Surfaces: Application Assessment as Rear Electrode for Silicon Heterojunction Solar Cells

Erkan Aydin[1,‡], Jehad K. El-Demellawi[2,‡], Emre Yarali[1], Faisal Aljamaan[1], Simone Sansoni[1], Atteq ur Rehman[1], George Harrison[1], Jingxuan Kang[1], Abdulrahman El Labban[1], Michele De Bastiani[1], Arsalan Razzaq[1], Emmanuel Van Kerschaver[1], Thomas G. Allen[1], Omar F. Mohammed[2], Thomas Anthopoulos[1], Husam N. Alshareef[2,], Stefaan De Wolf[1,*]*

[1]KAUST Solar Center (KSC), Physical Sciences and Engineering Division (PSE), King Abdullah University of Science and Technology (KAUST), Thuwal 23955-6900, Kingdom of Saudi Arabia.

[2]Physical Sciences and Engineering (PSE) Division, King Abdullah University of Science and Technology (KAUST), Thuwal 23955-6900, Kingdom of Saudi Arabia

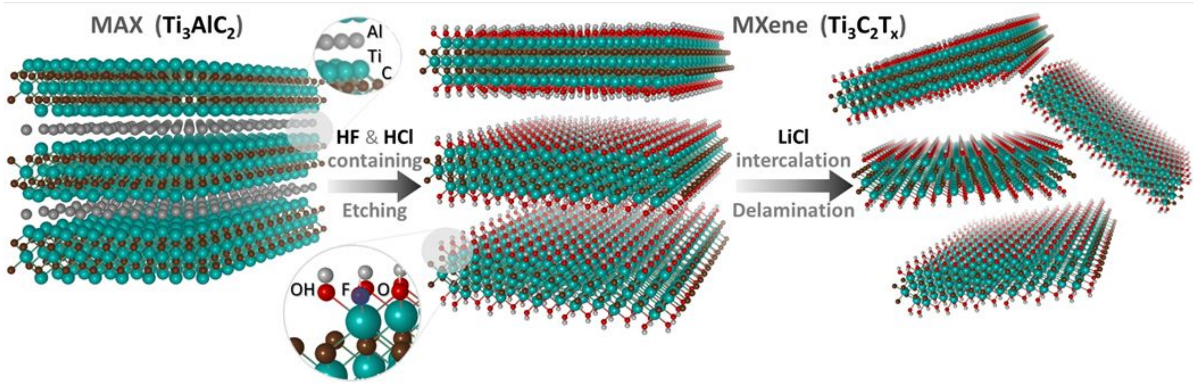

Figure S1. Schematic illustration for the synthesis pathway of $Ti_3C_2T_x$ in a fluoride- and chloride-containing etching bath followed by LiCl-based intercalation to obtain delaminated MXene flakes.

XPS Results and Data Analysis

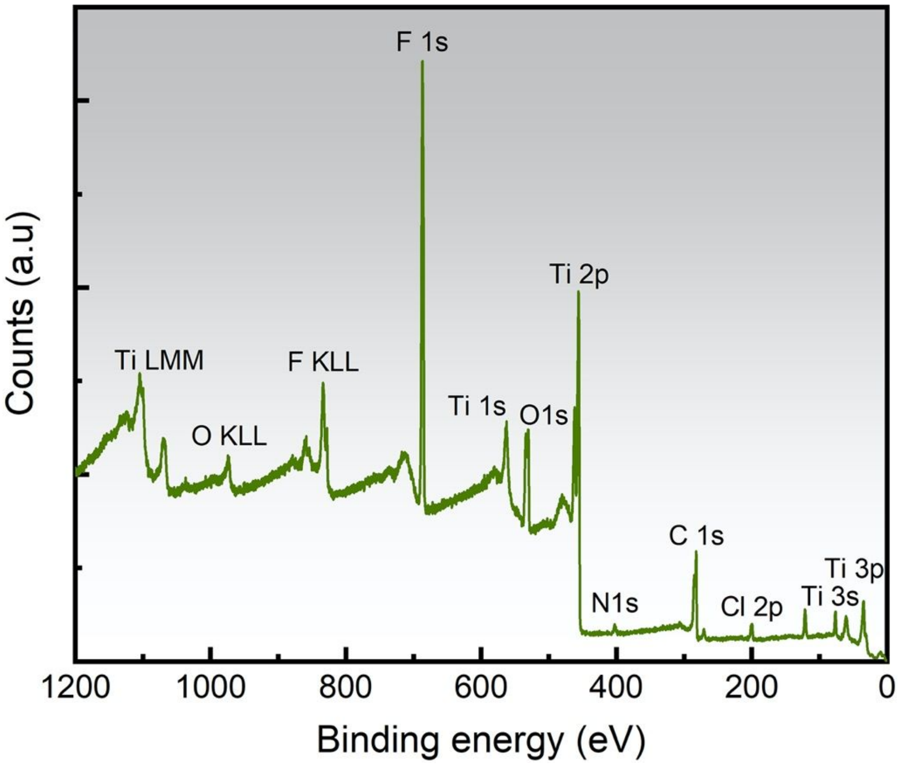

Figure S2. (a) XPS survey scan of a spray-coated $Ti_3C_2T_x$ film.

In accord with the detailed study of Halim et al.,[1] the envelope of the Ti 2p region was fitted, after background subtraction (using a Tougaard based function). Mixed Gaussian-Lorenzian GL(30) products were adopted with the metallic C-Ti-T_x asymmetry from the interaction of photoelectrons with the free electrons modeled with a tail (GL(30)T(1.1)) in correspondence to their work. The fits proceeded as follows: Initially, six doublets (12 components) were constrained by parameters detailed by Halim et al. These were limited to the FWHM (proportional to the C-Ti-T_x), binding energy positions (relative to C-Ti-T_x), and the spin-orbit splitting, *i.e.*, 6.2 for Ti-C and 5.6-6.3 eV for others. Finally, the intensity of the $p_{3/2}$, $p_{1/2}$ branching ratio was set equal to 2:1. Very minor modifications (<10%) were required to obtain satisfactory fits, with low residuals. The detailed fitting parameters are summarized below in **Table S1**. Following a similar fitting approach, the C 1s region was deconvoluted into six components, as shown in **Table S2**.

Table S1. The fitting parameters of the XPS Ti 2p core level. The values of the binding energies (BE) between brackets are for the $2p_{1/2}$ components.

**Peaks**	**Function**	**FWHM**	**BE** (eV)	**Spin Orbit Splitting** (eV)	**Relative Intensity**
**Ti**	GL(30)T(1.1)	0.953 (1.334)	455.51 (461.71)	6.2	18.03 (9.03)

**Ti^{2+}**	GL(30)	1.787 (2.621)	456.31 (461.71)	6.3	17.85 (8.94)
**Ti^{3+}**	GL(30)	2.501 (2.501)	457.71 (463.41)	5.7	22.07 (11.05)
**TiO_2**	GL(30)	1.072 (1.191)	459.112 (464.713)	5.6	1.51 (0.76)
**$TiO_{2-x}F_x$**	GL(30)	1.072 (1.191)	459.713 (465.713)	6	1.13 (0.56)
**$C-Ti-F_x$**	GL(30)	1.906 (3.34)	460.71 (466.71)	6	6.05 (3.03)

Table S2. The fitting parameters of the XPS C 1p core level.

Peaks	Function	FWHM	BE (eV)	Relative Intensity
**$C-Ti-T_x$**	GL(30)T(1.1)	0.608	282.275	41.62
**$C-C_{(aromatic)}$**	GL(30)	1.338	284.275	14.38
**$C-C_{(aliphatic)}$**	GL(30)	1.338	284.975	13.04
**C-O**	GL(30)	1.338	285.925	17.10
**C=O**	GL(30)	1.824	287.275	7.67
**O-C=O**	GL(30)	1.520	289.275	2.63

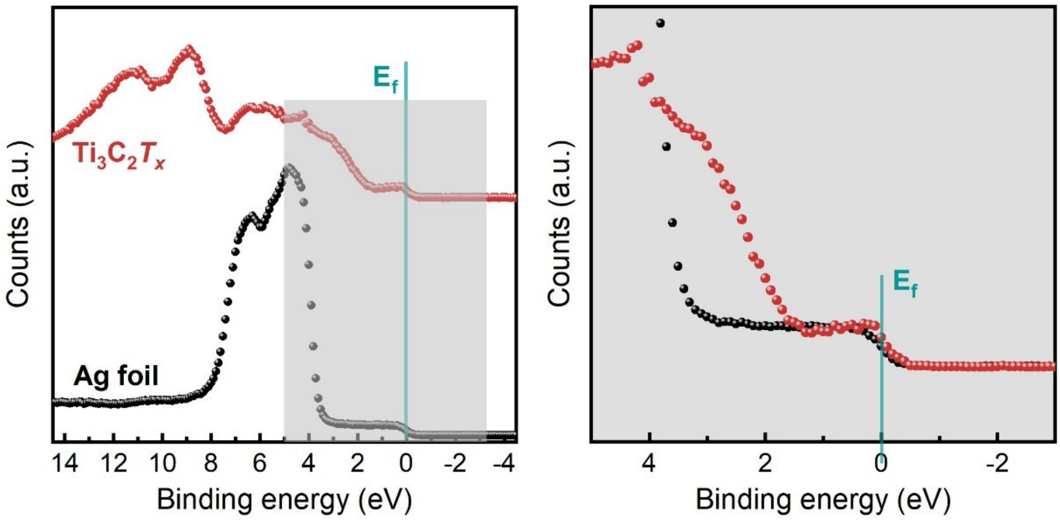

Figure S3. Valence band XPS (λν=1486.7 eV) spectra of $Ti_3C_2T_x$ (red) with a metallic (Fermi-Dirac) step feature, *i.e.*, qualitatively comparable with that of Ag (black). (left) Full spectra offset, (right) zoomed-in E_f region. This metal-like character is also confirmed by the large density of states (DOS) at the E_f, as demonstrated by UPS and IPES spectra in Figure 1g.

Ellipsometry Data Analysis

The spectroscopic ellipsometric data, Psi (ψ) and Delta (Δ), of the $Ti_3C_2T_x$/ITO/c-Si stack, were fitted at different incident angles (60º to 75º) to obtain the optical constants (n, k) and the complex permittivity (ε_1, ε_2) of a ca. 57-nm-thick $Ti_3C_2T_x$. The thickness and optical constants of the ITO layer in the $Ti_3C_2T_x$/ITO/c-Si stack were accurately determined using ellipsometry measurement on an ITO/c-Si stack (similar to the one on which $Ti_3C_2T_x$/ITO/c-Si was spray-coated), which was then used in the model of $Ti_3C_2T_x$/ITO/c-Si stack. The optical response of the $Ti_3C_2T_x$ film, as derived from the ellipsometric data were fitted with a Drude model and three Harmonic oscillators. The mean squared error (MSE) of the fitting and the experimental data is below 7. The corresponding fitting parameters are shown in **Table S3**.

Table S3. The fitting parameters of the optical response of a ca. 50-nm-thick $Ti_3C_2T_x$ film spray-coated on an ITO/c-Si stack.

Drude Oscillator [2]: $\varepsilon = \frac{-\hbar^2}{\varepsilon_0\rho_n(\tau_n \cdot E^2 + i\hbar E)}$			
ρ (Ω·cm)		τ (fs)	
2.46×10^{-4}		1.66	
Harmonic Oscillator [3]: $\varepsilon = \frac{[Amp_n + i(iAmp_n)]Br_n}{2}\left(\frac{1}{En_n - E - i\frac{Br_n}{2}} + \frac{1}{En_n + E - i\frac{Br_n}{2}}\right)$			
	Amp_n	Br_n (eV)	E_n (eV)
1st Harmonic	3.54	0.72	1.04
2nd Harmonic	3.82	0.56	1.63
3rd Harmonic	7.58	2.33	4.01

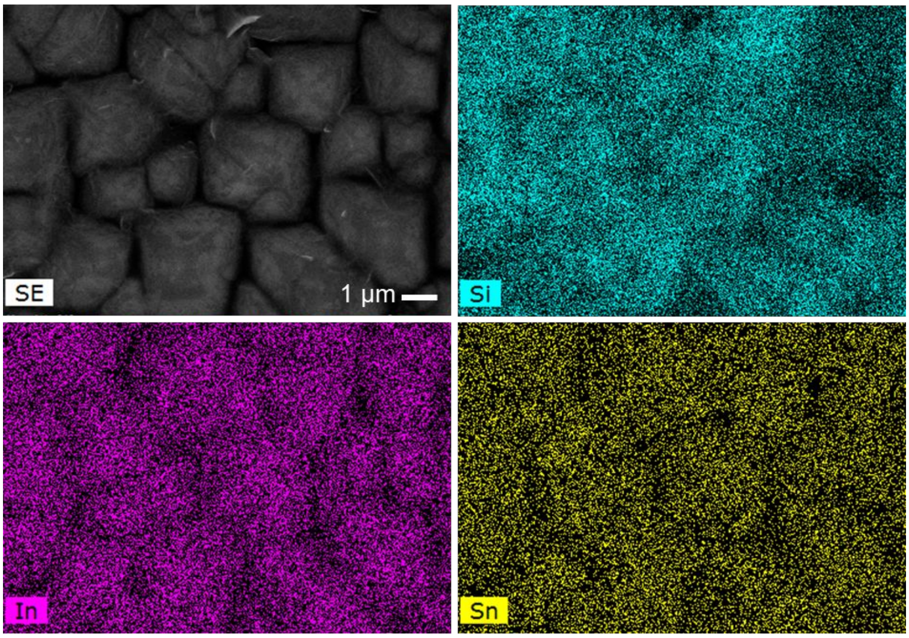

Figure S4. EDS mapping for the same top-view SE SEM micrograph depicted in Figure 3b showing the homogeneity of the Si (turquoise), In (purple), and Sn (yellow) across the $Ti_3C_2T_x$-contacted textured surface of the SHJ solar cells.

Table S4. Summary of the device characteristics (J_{SC}, V_{OC}, FF, and PCE) of the studied $Ti_3C_2T_x$-contacted SHJ solar cells (4.2 cm^2 in area) as a function of the number of spraying cycles. The optimum device performance was attained at 140 spraying cycles (red). The corresponding thicknesses were estimated using cross-sectional SEM and spectroscopic ellipsometry. For comparison, the concentration of the used MXene dispersions was fixed at ca. 1.5 mg/ml for all the batches of devices.

Spraying Cycles	40	60	100	140	160	260
$Ti_3C_2T_x$-contacted SHJs (4.2 cm^2)	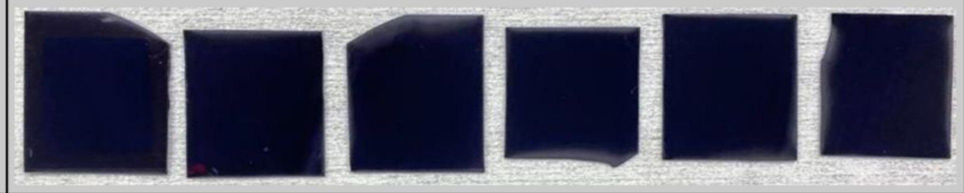					
Thickness (nm)	57	85	143	200	228	371
V_{OC} (mV)	699.9	714.5	710.8	710.3	708.8	706.8
J_{SC} (mA/cm^2)	35.8	27.4	37.2	37.4	37.5	37.2
FF (%)	64.1	72.2	71.5	74.4	72.6	71.9
PCE (%)	16.1	19.3	18.9	19.8	19.3	18.9

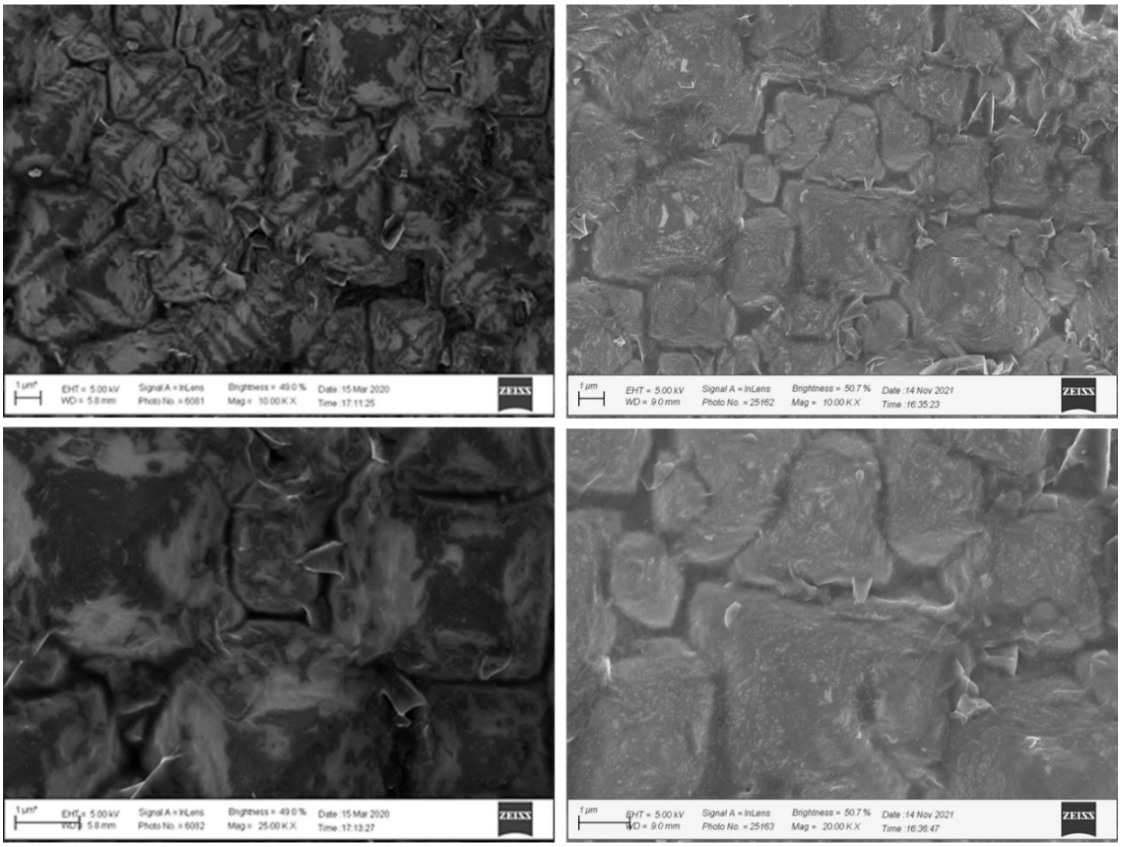

Figure S5. Top-view SEM micrographs of one of our MXene-contacted SHJ cells, pristine (left) and 609-days old (right). The prolonged ambient air storage induced no deterioration.

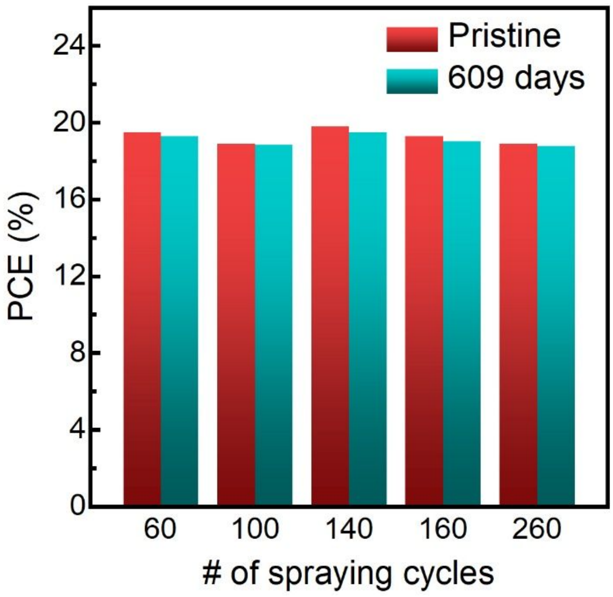

Figure S6. A statistical representation for the stability of different MXene-contacted SHJ solar cells (the same devices summarized in Table S4) after 609 days of ambient air storage. Compared to their pristine counterparts, all the 609-days old devices have retained ca. 99% of their initial PCE values, *i.e.*, 98.8, 99.7, 98.5, 98.7, and 99.5%, respectively, for those sprayed with 60, 100, 14, 160, and 260 cycles.

References

1. Halim, J.; Cook, K. M.; Naguib, M.; Eklund, P.; Gogotsi, Y.; Rosen, J.; Barsoum, M. W., X-ray photoelectron spectroscopy of select multi-layered transition metal carbides (MXenes). *Appl. Surf. Sci.* **2016,** *362*, 406-417.
2. Tiwald, T. E.; Thompson, D. W.; Woollam, J. A.; Paulson, W.; Hance, R., Application of IR variable angle spectroscopic ellipsometry to the determination of free carrier concentration depth profiles. *Thin Solid Films* **1998,** *313*, 661-666.
3. Adachi, S., *Optical properties of crystalline and amorphous semiconductors: Materials and fundamental principles*. Springer Science & Business Media: Boston, 2012.
